# Dendritic Guanidines as Efficient Analogues of Cell Penetrating Peptides

**DOI:** 10.3390/ph3030636

**Published:** 2010-03-12

**Authors:** Colin V. Bonduelle, Elizabeth R. Gillies

**Affiliations:** 1 Department of Chemistry, The University of Western Ontario, 1151 Richmond St., London, N6A 5B7, Canada; 2 Department of Chemical and Biochemical Engineering, The University of Western Ontario, 1151 Richmond St., London, N6A 5B9, Canada

**Keywords:** cell penetrating peptide, dendrimer, guanidine, drug delivery, cell-uptake, toxicity

## Abstract

The widespread application of cell penetrating agents to clinical therapeutics and imaging agents relies on the ability to prepare them on a large scale and to readily conjugate them to their cargos. Dendritic analogues of cell penetrating peptides, with multiple guanidine groups on their peripheries offer advantages as their high symmetry allows them to be efficiently synthesized, while orthogonal functionalities at their focal points allow them to be conjugated to cargo using simple synthetic methods. Their chemical structures and properties are also highly tunable as their flexibility and the number of guanidine groups can be tuned by altering the dendritic backbone or the linkages to the guanidine groups. This review describes the development of cell-penetrating dendrimers based on several different backbones, their structure-property relationships, and comparisons of their efficacies with those of known cell penetrating peptides. The toxicities of these dendritic guanidines are also reported as well as their application towards the intracellular delivery of biologically significant cargos including proteins and nanoparticles.

## 1. Introduction

In recent decades there has been significant interest in the preparation of new materials for the delivery of drugs and imaging agents. This interest is motivated mainly by the problematic properties of these molecules. For example, while genomics and proteomics are revealing many promising drug candidates based on peptides and oligonucleotides, these molecules are often unable to reach their target sites within cells as they are not capable of traversing cell membranes. On the other hand, cell penetrating peptides (CPPs) are a class of molecules that exhibit the ability to cross biological barriers. They were discovered from serendipitous observations on the HIV-1 Tat trans-activating factor and on the *Drosophila Antennapedia* transcription factor [1,2,3,4]. Since then, the search for other CPPs has been ongoing, either by the screening of natural proteins or by rational design [5,6,7,8,9,10,11,12,13,14,15,16]. 

The functions common to all CPPs include their abilities to rapidly enter cells and their high water solubilities [17]. While the main classes of CPPs differ from one another in structure [17,18] (Table 1), they are generally composed of large fractions of basic amino acids and in particular often contain a high concentrations of guanidinium moieties [18,19,20,21,22,23]. Indeed, the translocation capabilities of CPPs are strongly influenced by the presence of positively charged residues, particularly arginine [13,14,15,24]. The observation of this primary structure-function relationship has thus far led to the development of 1) peptide analogues where residues are replaced by positively charged ones such as arginine [13,23,24,25] and 2) nonpeptide analogues that contain positively charged functionalities including guanidine groups [23,24,26,27,28,29,30,31,32].

**Table 1:** Amino acids sequences of the main CPPs.

**Tat49-57**	RKKRRQRRR
**Polyarginine**	RRRRRRRRR
**Decalysine**	KKKKKKKKKK
**Penetratin**	RQIKIWFQNRRMKWKK
**Transportan**	GWTLNSAGYLLGKINLKALAALAKKIL
**MPG**	GALFLGFLGAAGSTMGAWSQPKKKRKV
**Pep1**	KETWWETWWTEWSQPKKKRKV

Several studies have been carried out in order to elucidate the role of the arginine residues in the HIV Tat_49-57 _(RKKRRQRRR) sequence [33]. For example, the replacement of each residue in this sequence by alanine showed that arginine and lysine residues were essential to the cell-uptake [24]. When the non-charged glutamine was replaced by an alanine (RKKRRARRR), cell-uptake was less affected. The conclusion was that positive charge was important for uptake. Subsequently, it was found that a lysine 9-mer (KKKKKKKKK) performed less well than the original Tat_49-57 _sequence. In marked contrast, the corresponding arginine 9-mer (RRRRRRRRR) was superior to the Tat reference [24]. These studies therefore showed an obvious link between translocation and arginine residues. Experiments have also been conducted to investigate the effects of oligomer length. Studies on the HIV Tat_49-57 _sequence revealed that truncations led to less effective uptake [24]. In a study on oligoarginines comprising 6–20 arginine residues, a maximum activity was found for the 15-mer [13,34]. For cost reasons concerning synthesis, the 8-mer (RRRRRRRR) was chosen by Wender and coworkers [35] but this number also seemed to be the most efficient in several other cases [36]. 

The interesting finding that oligomers comprising either all *D* or all *L* amino acids worked similarly suggested that the backbone of the transporter was not critical to uptake [13]. Indeed it has been shown that interdigitation of an arginine backbone with α, β, γ, or δ amino acids [25] or even replacement of the peptide backbone with an oligocarbamate [30] improved cellular uptake relative to the corresponding arginine oligomers. By investigating different backbones, it was shown that the main backbone requirement for cellular uptake of arginine-rich peptides was conformational freedom, an essential parameter to afford an optimized interaction with the cell membrane moieties [33]. 

Overall, research on various linear analogues of CPPs has revealed the importance of multiple guanidine groups and the possibility to vary the backbone and the spacing between guanidines without loss of cell-uptake efficiency [23,25,30,37,38]. As a next step, if CPPs are to be used to deliver biologically active cargoes into cells, another important design consideration should be their ease of synthesis. While peptides are often prepared by iterative syntheses on solid supports, the preparation of large quantities is still quite costly and may prove limiting for the widespread pharmaceutical application of these materials. The preparation of other non-peptide linear oligomers by convergent solution syntheses also involves many synthetic reactions and purification steps [23,24,25,30]. Therefore, the development of cell penetrating molecules that can be efficiently prepared on larger scales and which can be readily conjugated to their cargos would represent an important step towards extending their utility in drug delivery applications. Dendrimer synthesis, once optimized, can be efficient and cost-effective, as indicated by the increasing numbers of dendrimers available commercially on a multi-gram scale. For example, one can purchase 2 g of a 3rd generation polyamidoamine (PAMAM) dendrimer for less than US $400 and functionalize it with guanidines in a single step. In contrast, for a similar price one can only purchase less than 10 mg of the Tat_49-57_ peptide (depending on the supplier). In addition, as the backbones are tunable their flexibilities can be optimized [39,40]. Furthermore, dendrimers can be designed to be resistant to rapid biodegradation, unlike many linear peptides [41,42,43,44]. Because of these potential advantages, dendrimers represent promising backbones for the display of multiple guanidine residues. This review is focused on the use of dendritic guanidines as analogues of cell-penetrating peptides. It is an attempt to summarize the state-of-the-art in this field. The following sections will successively discuss the unique properties of dendrimers, their syntheses and functionalization with guanidine groups, the cell penetrating capabilities of the resulting dendritic guanidines, their structure-property relationships and their conjugation to cargo.

## 2. Dendrimers as Cell-Penetrating Agents

**Figure 1:**
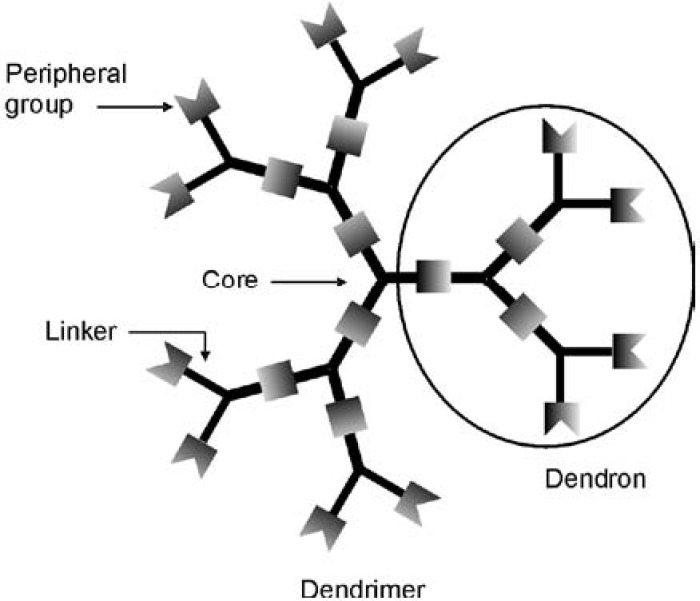
Schematic of a dendrimer structure.

While peptides are typically linear macromolecules, highly branched structures are also found widely in nature, where the branching and display of multiple terminal functionalities enable the enhancement of functions. At the interface of polymer and synthetic organic chemistry, “dendrimers” have emerged over the past couple of decades as a new class of highly branched molecules. Named based on the Greek word *dendron*, meaning “tree”, dendrimers are macromolecules that have structures resembling those of trees (Figure 1). As dendrimers are synthesized from branched monomer units in a stepwise manner, monodisperse products are often obtained. In addition, it is possible to achieve precise control over their molecular shape, dimensions, density, polarity, flexibility and solubility by choosing different building/branching units and surface functional groups [45,46,47,48,49]. Generally, dendrimers are globe- or ellipsoid-shaped, and they consist of three distinct regions: (1) a central core, (2) repeated branches, and (3) surface functional groups [50]. The central core should be a molecule with at least two reactive functional groups. The repeated branches are organized in a series of radially concentric layers called ‘’generations’’ [51]. When iterative growth is conducted from focal point or core molecules containing one reactive group, wedge-shaped structures, commonly referred to as “dendrons” result. 

Dendritic structures have been recognized to be ideal building blocks for biomedical applications because of their monodispersities, high loading capacities, large-scale production, and bioconjugation capabilities [39,52,53,54]. Most pertinent to their development as analogues of CPPs, the properties of dendrimers are often dominated by their peripheral groups, particularly at high generations owing to the exponential increase in the number of peripheral units with each generation. Thus dendrimers may serve as useful backbones for the conjugation of multiple guanidine groups. Tuning the dendrimer or dendron generation allows the number of guanidine groups to be controlled, while the distance between gaunidines and the flexibilities of the structures can be determined by the selection of the branching monomers and linkers.

**Figure 2:**
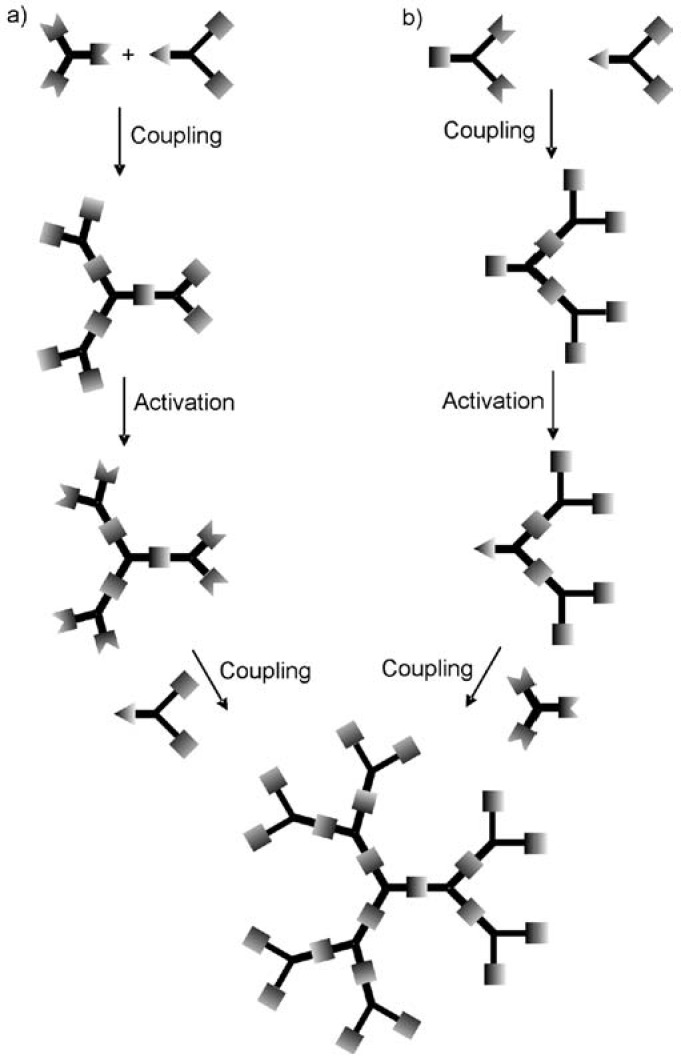
Comparison of the (a) divergent and (b) convergent approaches to dendrimer synthesis.

Generally, there are two approaches used to synthesize dendrimers: the divergent method and the convergent one [40,55]. In the divergent approach, the dendrimer is grown outwards from the core by the repetition of coupling and activation steps as shown in Figure 2a. Repetition of these coupling and activation steps provides an exponential increase in the number of peripheral groups, and reactions at each step. In the convergent approach (Figure 2b), growth initiates from what will become the dendrimer periphery and progresses towards the core. First the peripheral groups are coupled to each branch of the monomer, while keeping the focal point of the monomer in an unreacted form. This focal point can then be activated and subsequently coupled to another monomer unit. This reaction sequence of coupling and activation continues until the desired generation is reached, and then the resulting dendritic fragments, referred to as “dendrons” are finally coupled to a core molecule. The convergent approach often affords dendrimers with perfect structural homogeneity due to the small number of coupling reactions performed at each step and the possibility to remove flawed structures by purification methods such as chromatography [51,55,56]. However, the divergent approach is the preferred one for the large scale industrial preparation of dendrimers because the quantity of dendrimer sample increases with each generation and the removal of excess reagents by techniques such as precipitation, distillation, or ultrafiltration is facilitated by their differences in mass. In some cases, a divergent synthesis can yield a monodisperse product [57,58,59]. In other cases minor structural flaws resulting from incomplete couplings or side reactions can exist, [47,60] but the polydispersities are still as low or lower than can be achieved in the best controlled polymerizations. Although the focus of this review is not to describe the syntheses of the dendrimer backbones in detail, these approaches have allowed for the preparation of several families of dendrimers such as the PAMAM [61], polypropyleneimine (PPI) [62], polyester [63,64,65,66], amino acid [67,68,69,70,71], carbohydrate [72,73] and various hydrophobic dendrimers [74,75]. At this stage, the syntheses of dendrimers is a sufficiently well developed field such that many dendrimer backbones can be prepared on a multigram or larger scale, and several dendrimer backbones including the PAMAM, PPI, polyesters, and polylysines are even commercially available. The ready availability of these backbones provides an additional advantage in their development as analogues of CPPs.

## 3. Syntheses of Guanidine Functionalized Dendrimers

**Figure 3:**
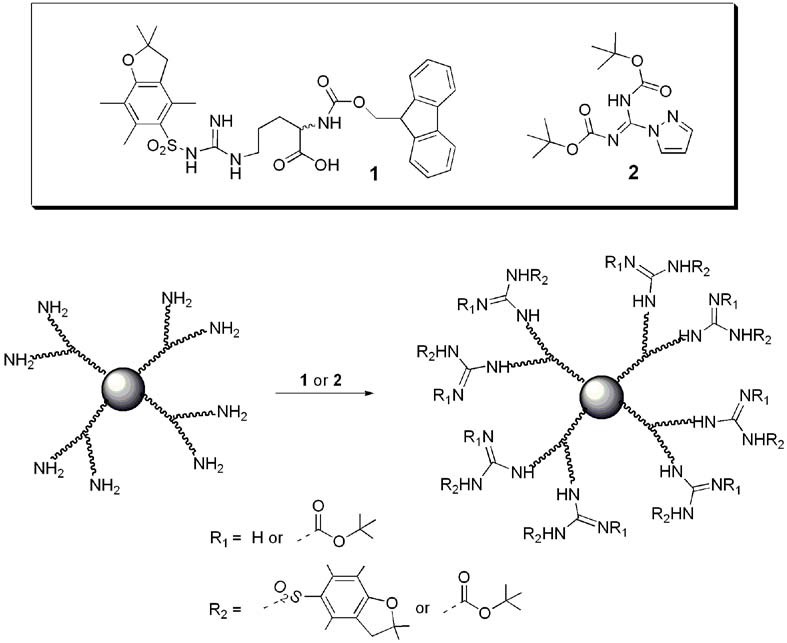
Introduction of the guanidine functionality to a dendrimer’s peripheral amine groups using Fmoc protected arginine (**1**) or 1H-pyrazole-1-carboxamidine (**2**).

With many diverse dendrimer backbones readily available commercially or through chemical synthesis, the guanidine end group is generally introduced after the dendrimer synthesis by a final functionalization step. This usually involves guanidinylation of terminal amino groups on the dendrimer using either fluorenylmethyloxycarbonyl (Fmoc) protected arginine (1) [76] or 1*H*-pyrazole-1-carboxamidine (**2**) [77] (Figure 3). The guanidine moiety of these molecules is generally introduced in a protected form and the protecting group is removed from the dendrimer in the final step.

**Figure 4:**
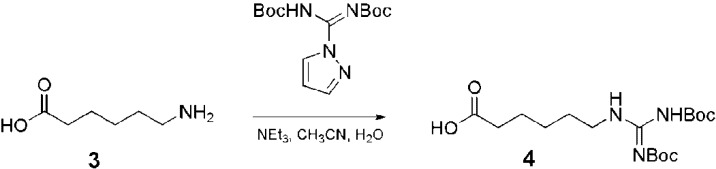
Synthesis of a guanidine functionalized precursor having a carboxylic acid moiety for coupling to a polyester dendrimer periphery [94].

Fmoc-protected arginine has been used to functionalize polyamide based dendrimers [78,79,80,81,82,83,84] (generally PAMAM) [79,80,81,82,83,84]) and PPI dendrimers [85]. The pyrazole carboxamidine represents an efficient alternative to increase the reactivity of the coupling and was used to functionalize polyamide [29,86], PPI [87,88,89,90,91], melamine [92] and polyether based dendrimers [93]. In the case of polyester based dendrimers [94], pyrazole carboxamidine **2** was used to create a guanidine functionalized precursor with a carboxylic acid moiety **4** that was then coupled to the polyester dendrimer by using carbodiimide chemistry (Figure 4).

**Figure 5:**
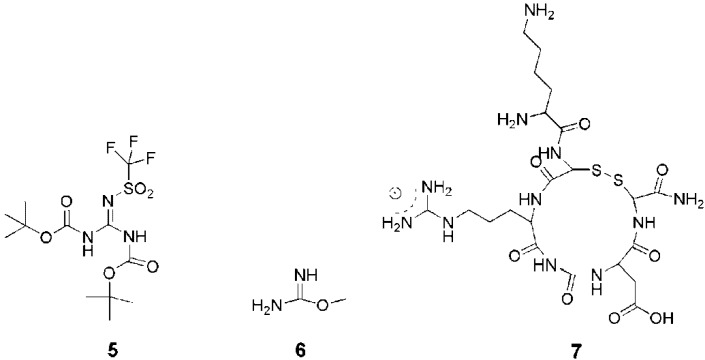
Precursors for the introduction of guanidine moieties.

Occasionally, other precursors such as *N,N’*-diBoc-*N’’*-triflylguanidine (**5**) [26,95,96,97], *O*-methyl-isourea hydrochloride (**6**) [98,99] or an arginine-containing tri/tetrapeptide **7** [100,101,102] (RGD or CRGD to target αvβ3 Integrins) have also been used to introduce guanidine moieties (Figure 5).

## 4. Structures and Cellular Internalization of Guanidine Functionalized Dendrimers

The translocation of a branched-chain arginine transporter was first investigated with success by Futaki *et al*. [27]. It was shown that peptide clusters formed by approximately eight arginine residues played a crucial role in translocation. Moreover, flexibility in the positions of the positive charges in these peptides confirmed that a continuous arrangement of the arginine residues was not required. As described above, dendrimers are promising alternative branched backbones for the multivalent display of guanidine functionalities due to their relatively economical syntheses and abilities to display varying numbers of guanidine moieties in a controlled manner. 

**Figure 6:**
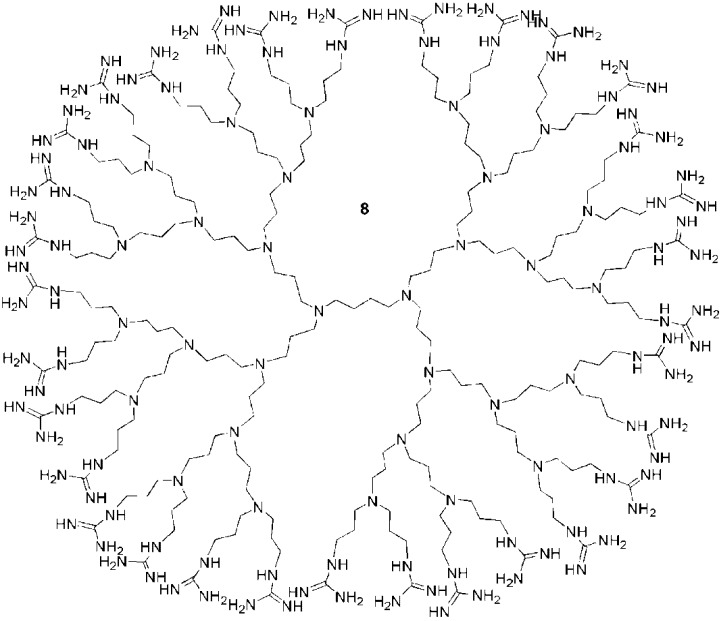
A guanidine functionalized 4th generation PPI dendrimer [89,90].

Initially, guanidine functionalized PPI dendrimers such as **8** (Figure 6) were investigated as analogues of CPPs by Paleos and coworkers. The first studies on these macromolecules were focused on liposome internalization of 4th and 5th generation dendrimers [89,90]. With a 4th generation dendrimer, liposome uptake was enhanced by the number of guanidine groups. For example, for 0, 6, or 12 guanidines on the dendrimer surface, the liposome uptake was 25%, 60% and 80% respectively. Moreover, for equal concentrations of guanidine moieties, the higher generation dendrimers proved to be more effective in interacting with liposomes. This behavior was attributed to the multivalent effect of the dendrimer [103,104]. Nevertheless, this interaction enhancement with increasing dendrimer generation was not correlated with liposome penetration efficiency. An investigation of 3rd and 4th generation guanidine functionalized PPI dendrimers bearing either 16 or 32 guanidine groups respectively showed that the lower generation dendrimer was more effective in penetrating the liposomes [87]. It was concluded from this work with liposomes that the constraints imposed by the size and the dense surface functionalization inhibited effective liposome internalization of the higher generations despite the multivalent effect leading to a stronger interaction. The effects of decorating the surface with other functionalities were also tested. For example, guanidine functionalized PPI dendrimers were partially actetylated [87] or partially hyroxylated [88]. On A549 lung carcinoma cells, partially acetylated derivatives of the 3rd and 4th generation dendrimers showed enhanced translocation abilities compared to the non-acetylated derivatives, an effect attributed to their increased hydrophobicities. In this case, the number of guanidines, eight for the 3rd generation and 14 for the 4th generation, was crucial. Cell uptake was localized to the nucleus for the 3rd generation dendrimer and to the cytosol for the 4th generation dendrimer [87]. The introduction of hydroxyl groups was accomplished by the ring opening of propylene oxide and led to a decrease in internalization of a 4th generation dendrimer in HEK 293 and COS-7 cells [88]. However, in this latter case, the partially hydroxylated dendrimers were less toxic.

**Figure 7:**
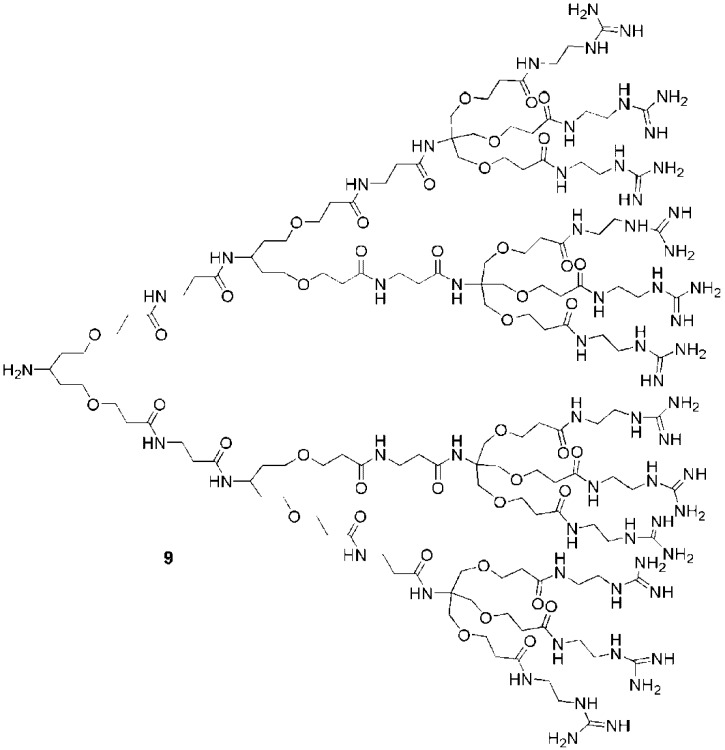
A polyamide dendrimer with 12 peripheral guanidines [26].

The second backbone investigated for the development of guanidine functionalized dendrimers was a polyamide. The first studies on these dendrimers were performed by Goodman and coworkers who synthesized dendrons with focal point amine groups for coupling to cargos [26]. These dendrons such as **9** (Figure 7) were internalized efficiently into HeLa S3 cells as well as human cervical carcinoma cells. Dendrimers with different numbers of guanidines (1, 3, 6, 9, or 12) on their surfaces were tested. The dendrimers bearing 6, 9 or 12 guanidines exhibited better uptake than dendrimers with 1 or 3 guanidines, suggesting that 6 guanidine groups were sufficient to achieve good translocation. A comparative assay with Tat_49-57_ conjugates revealed that dendrons with 9 guanidine groups exhibited the same cell penetrating capabilities as Tat_49-57_. Nevertheless, the dendron was not able to cross the nuclear membrane as efficiently as the Tat_49-57_ conjugate. Although the presence of a fully complexed Tat_49-57_-chromophore in the nucleus had not been demonstrated in this work, it has since then been elegantly confirmed by using Cre recombinase as cargo that the Tat conjugate reaches the nucleus without compromising the survival and competency of the cells [105,106].

**Figure 8:**
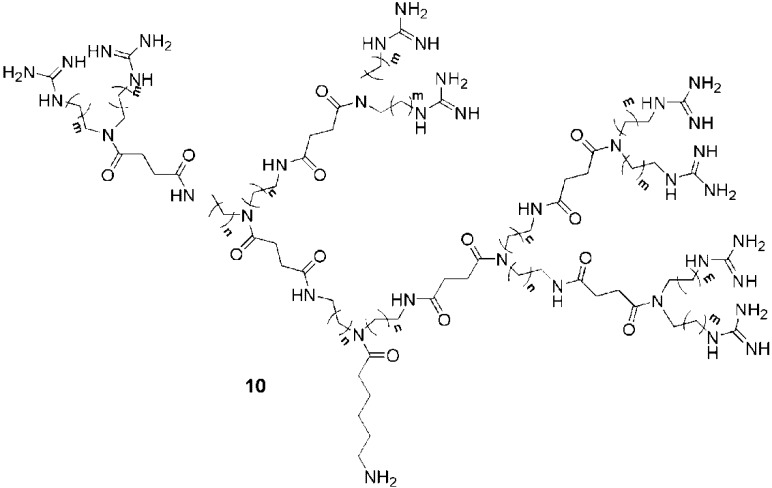
Guanidine functionalized polyamide dendrimers incorporating difference spacers (*n,m = 1,2 or 5*) [29].

In other work, the prospect of new guanidine-based transporters prompted Wender and coworkers to study tunable guanidine functionalized polyamide dendrimers [29]. These authors prepared a series of 9 dendrons, all bearing 9 guanidine groups on their peripheries (Figure 8). Tunable spacers (n or m, see Figure 8) incorporated throughout the dendron backbones permitted a study of the effect of spacer length on cell uptake efficiency in the human T cell line Jurkat. The results revealed that the longest hydrocarbon spacers led to faster rates of uptake into cells. For dendrons such as **10** (n = 5, m = 2 or 5), the efficiencies exceeded those of the linear oligoarginine transporter (9-mer). The increased uptake of the dendrons with the longer spacers may be attributed to the increased flexibilities of the backbones as was observed for the linear CPPs; however, the incorporation of longer spacers also introduced increased hydrophobicity which may have enhanced the interaction of the molecules with membranes and thus their uptake. In order to separate the effects of flexibility and hydrophobicity, it would be necessary to compare molecules having hydrophilic versus hydrophobic spacers.

**Figure 9:**
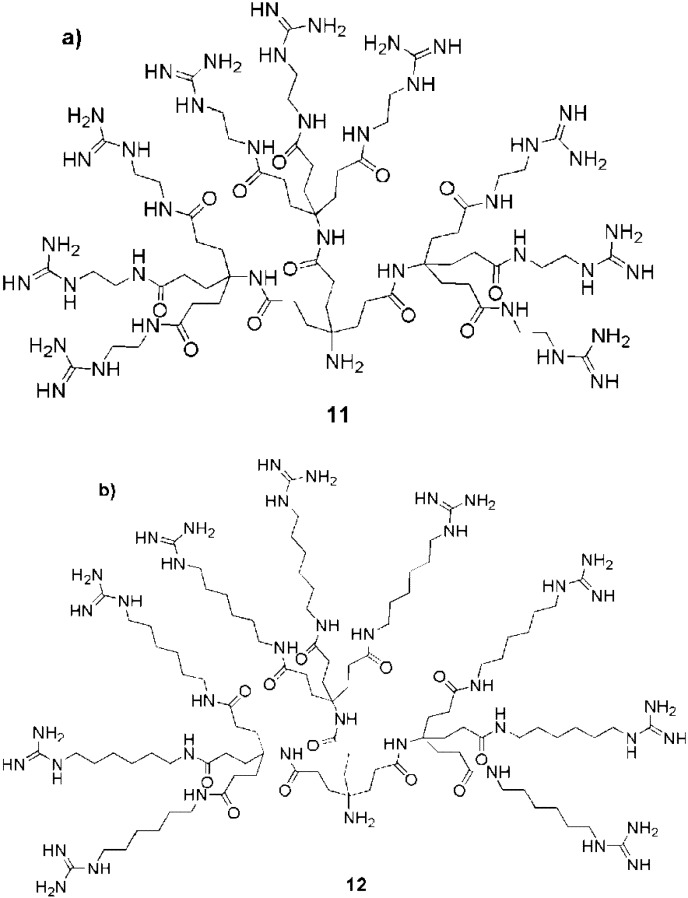
Guanidine functionalized dendrimers having either (a) C2 or (b) C6 spacers [95].

The effects of spacers were further investigated by Harth et al. They prepared two different dendrons, each bearing 9 peripheral guanidines but with two different spacers: C2 length (**11**) or C6 length (**12**) (Figure 9) [95]. Fluorescent conjugates of these dendrons were tested with NIH-3T3 and HMEC cells and the conjugate with the longer spacer was internalized more rapidly. 

Moreover, while the dendron with the longer spacer was localized in the cell nuclei after internalization, the other conjugate was taken up into cytosolic compartments. Dendron **12**, containing the C6 spacer was also used to create protein conjugates [96,97] but the nuclear localization observed with the dendron alone was lost in these cases. 

**Figure 10:**
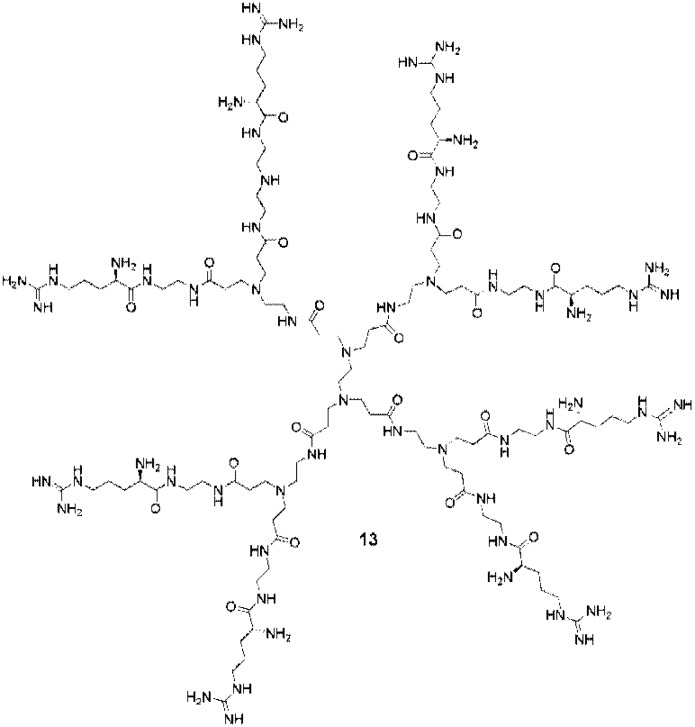
A PAMAM dendrimer functionalized with guanidine groups [85].

Another polyamide-based dendrimer investigated for cell penetration was the well-known PAMAM dendrimer. The first attempt to functionalize PAMAM dendrimers with guanidine groups was performed by Park and coworkers [80]. These authors compared the transfection efficiencies of an arginine functionalized PAMAM dendrimer **13** (Figure 10) with a lysine functionalized PAMAM dendrimer. 

The results obtained using 293, HepG2, Neuro 2A and vascular smooth muscle cells strongly evidenced the efficiency of the guanidinylated dendrimer and its potential as a good nonviral gene carrier. This first study, carried out using a 4th generation dendrimer with 58 peripheral guanidines opened doors to vulnerable-cell transfection [79]. Further studies on the 4th generation dendrimer were also done by the same group to evaluate its ability to cross monolayers of CaCo-2 cells [81]. The results suggested a paracellular transport mechanism (apical-to-basolateral or basolateral-to-apical) revealing the potential for these macromolecules to be used in oral absorption applications.

**Figure 11:**
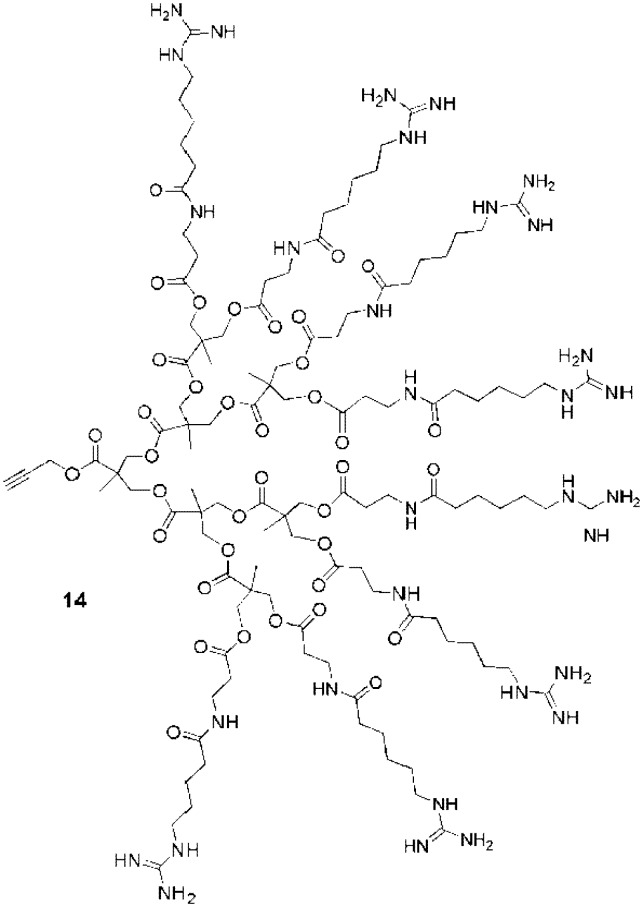
Guanidine functionalized polyester dendron with a ‘clickable’ focal point [94].

**Figure 12:**
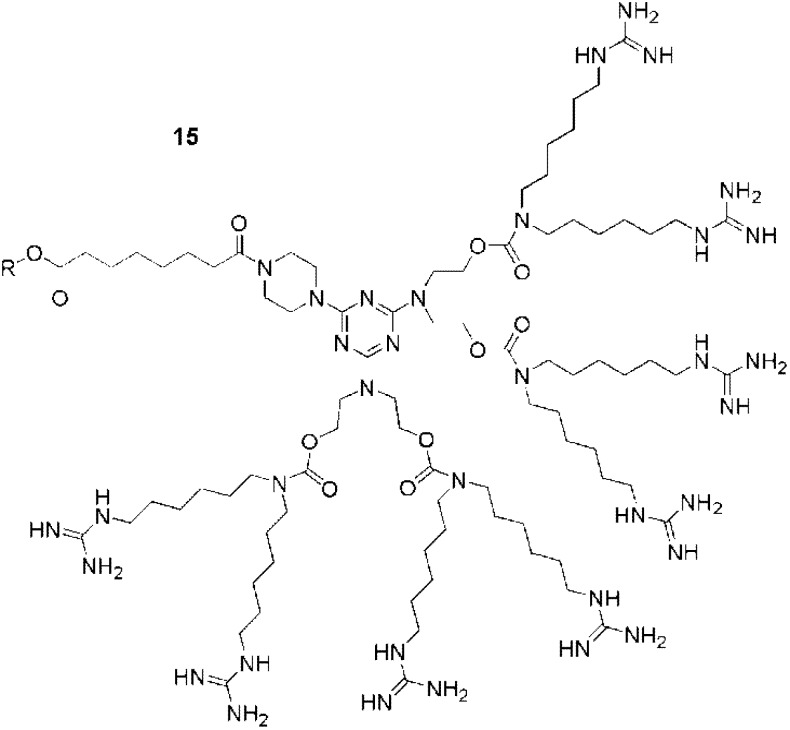
Guanidine functionalized polycarbamate dendron [98,99].

Two other guanidine functionalized dendrimers were also developed for cell penetration. A 3rd generation polyester dendron (**14**, Figure 11) bearing 8 peripheral guanidines exhibited cell-penetrating efficiencies similar to those of Tat_49-57 _in GL261 mouse glioma cells [94]. Interestingly, this dendron presents a clickable propargyl group at its focal point for conjugation to cargo. The second example was a polycarbamate-based dendron (**15**, Figure 12), also bearing 8 guanidines. Coupled with different cargos, this CPP analogue gave efficient transfection results *in vivo* (in mice) [98,99]. From the studies described above, it is evident that guanidinylated dendrimers are able to act as CPP analogues, with efficiencies similar to those of the peptide Tat_49-57_. A wide variety of dendrimer backbones have been functionalized with guanidine moieties, and the effects of the linkers, dendrimer backbones, and the numbers of guanidines have also been investigated. As for CPPs, these studies have suggested the importance of the flexibilities and conformational freedom of the guanidine moieties in the molecules. With dendrimers, this freedom was easily achieved by tuning the length of the spacers between the guanidine groups and the dendrimer peripheries. It should also be considered that the lengths of these spacers may also affect the overall hydrophobicities of the structures, which may in turn affect their uptake. Furthermore, it was also shown that the structures of the dendrimers impacted the cellular localization (cytosolic *vs.* nuclear), thus suggesting that dendrimers as CPPs analogues are also useful to cross different types of biological membranes.

**Table 2:** Guanidinylated dendrimers used for cell-uptake studies.

Backbone	Guanidines	Cargo	Cell-line	Reference
Poly(propylene imine)	0,6,12	Betamethasone derivatives	Liposomes	[89]
Poly(propylene imine)	4,8	-	Liposomes	[90]
Poly(propylene imine)	8,14	FITC	Human lung carcinoma (A549)	[87]
Poly(propylene imine)	16,32	-	Liposomes	
Poly(propylene imine)	0-32	DNA	HEK293, COSY7	[88]
Poly(propylene imine)	8	DNA	HeLa, 293 cells	[85]
Polyamide	3,6,9,12	GFP, FITC	HeLa S3 and Human cervical carcinoma	[26]
Polyamide	9	FITC	NIH-3T3 fibroblasts and HMEC	[95]
Polyamide	9	IgG-Antibodies	Hep-2 (RSV-GFP)	[97]
Polyamide	9	Polymer nanoparticles	NIH-3T3 fibroblasts	[96]
Polyamide (dendrigrafts)	12	FITC	Human lung carcinoma (A549)	[86]
Polyamide	8	FITC	Human lymphocyte (T cell line Jurkat)	[29]
PAMAM	58	DNA	293, HepG2, Neuro2A, rat vascular smooth muscle cells	[80]
PAMAM	58	DNA,RNA	Primary cortical cells (Neurons, glial cells)	[79]
PAMAM	16,32,64	DNA,RNA	293, HUVECs	[81]
PAMAM	64	DNA	HepG2, HeLa, SMCs, HUVECs	[82]
PAMAM	31,59,60,116	DNA	HeLa, 293, A549	[83]
Polycarbonate	8	Oligonucleotides	mouse	[99]
Polycarbonate	8	Oligonucleotides	mouse	[98]
Polyester	8	Fe_3_O_4_ nanoparticles	GL261 mouse glioma	[94]

## 5. Cellular Internalization Mechanism

CPPs can enter cells through multiple mechanisms. Theoretically, both endocytosis and diffusion to the cytosol can occur. Wender highlighted that for CPPs a universal uptake mechanism is not very probable [23]. However, he defined two common aspects of any mechanistic possibilities derived from the guanidine group: an association occurs first and is followed by the cell uptake itself. 

The first association step involves ionic and hydrogen bonding interactions between the negatively charged carboxylates, phosphates and sulfates belonging to membrane constituents and the guanidine groups, which are protonated and thus positively charged at physiological pH. It was shown that the bidentate hydrogen bond network formed with guanidinium groups is particularly strong and important. For example, monomethylation or dimethylation of the guanidine groups strongly affected the cell uptake with decreases of 80% and 95% respectively [107]. Moreover, the spatial orientation of the guanidiniums was another key point to facilitate the interaction with the membrane. Space and freedom were required because negatively charged functionalities on the membrane diffuse and repel each other [23,24]. Also of importance were the organizational [108] and multivalent effects [103,104] that act synergistically to enhance the binding.

After the first association step, cell-uptake occurs. Theoretically, either endocytosis or diffusion processes permit molecules to enter cells. It is generally accepted that diffusion only occurs for small molecules (molecular weight < 3,000 g・mol^-1^) and involves passive or active transport through the membrane, depending on the molecule [109]. It was shown that endocytosis is certainly involved for CPPs, because at 4 ºC, a temperature known to inhibit endocytosis, a significant decrease is generally observed [110,111]. It is important to note that some uptake is observed under conditions that inhibit endocytosis, suggesting that simple diffusion probably also occurs [13,112,113]. Numerous models for understanding how this direct diffusion of CPPs could occur have been suggested as an inverted micelle model [3], a carpet model [114] or an adaptive translocation model [107]. More details concerning the endocytosis/direct diffusion of CPPs can be found in recent reviews by Wender et al. [23] and Nakase et al. [115]. 

**Figure 13:**
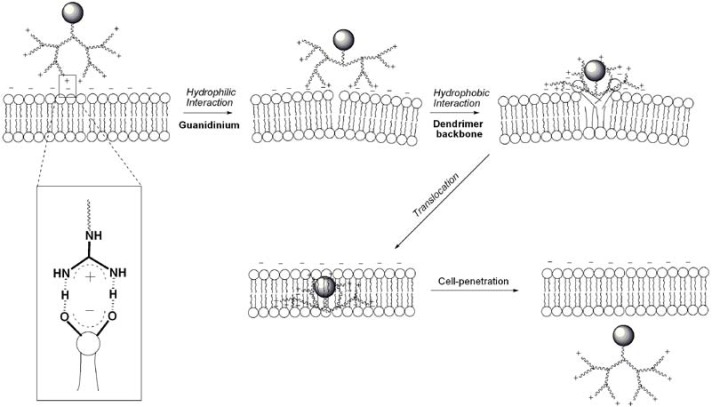
Postulated mechanism of direct cell penetration for a guanidine functionalized dendrimer/dendron.

For dendrimers, all of the elements required for strong interactions between the guanidiniums and the negatively charged membranes were found to be important, including the use of flexible spacers for conformational freedom, and the incorporation of multiple guanidines for organizational and multivalent effects [116]. For guanidine functionalized dendrimers, Paleos and coworkers proposed a mechanism based on liposome interaction studies that is in principle, applicable to cells [28] (Figure 13). This mechanism is based on the possibility for dendrimers to exhibit adaptive solubility behaviour [117,118], the ability to become hydrophobic or hydrophilic depending on the environment. After charge neutralization, a change in the dendrimer’s conformation could occur, leading to the exposure of the dendrimer’s hydrophobic interior to the interior of the cell membrane. By this mechanism, the dendrimer may be translocated easily by simple diffusion. This mechanism is certainly concomitant to endocytosis, which may be more important if large or highly hydrophilic cargo is attached to the dendrimer.

## 6. Toxicity of Guanidine Functionalized Dendrimers

One drawback of the use of polycationic vectors is their potential cell toxicity [119,120]. It is important to highlight that toxicity is a relative concept, strongly dependent on the concentration used. Moreover the toxicity as well as the cell-uptake efficiency are cell-line dependent.

There are several recent examples of the use of CPPs as therapeutic vectors, in which they were found to be well tolerated *in vivo* [7,121,122,123]. Nevertheless, CPPs have also revealed potentially toxic behaviour [124,125,126]. For example, the full-length Tat protein exerted a toxic action on primary rat neuronal cultures, inducing neuronal cell death that was correlated with the time of exposure [127]. The neurotoxic effects of this Tat protein had also been previously revealed [128] as well as for the basic region Tat_49–57_ alone [129]. This basic domain was also able to induce endothelial cell apoptosis [130]. Although at 100 μM Tat_48–60_ did not induce any significant toxicity during a period of 24 h on HeLa cells [4], at concentrations as high as 1 mM, Tat_49–57_ demonstrated a toxic effect in all the cell lines tested [131]. The precise mechanisms leading to this cytotoxicity still remain unclear but the membrane-disrupting potential of CPPs appears to be correlated with the hydrophobic moment of the peptides [125]. Moreover, the toxicity of CPPs depends heavily on the peptide concentration, on the cargo molecule attached and on the coupling strategy used [126].

First, it is important to emphasize that there is nothing inherently concerning about dendrimers or dendrons. Dendrimers are increasingly being used in a wide variety of biomedical applications [132,133] where a number of dendrimer backbones have been found to be well tolerated. For example, polyester dendrimers have been investigated as carriers of the anticancer drug doxorubicin, and even at doses higher than that of the free drug, the delivery system was less toxic [134]. In addition, PAMAM dendrimers functionalized with the anticancer drug methotrexate and folic acid as a targeting group have been found to be well tolerated *in vivo* [135]. Another significant example is the dendrimer-based microbiocide VivaGel^TM^, which is in clinical trials for the prevention of HIV or HSV-2 (genital herpes) transmission [136]. On the other hand, polycationic polymers and dendrimers such as PAMAM and poly(ethyleneimine) (PEI) have also been demonstrated to be toxic under some circumstances. They can induce the formation of nanoscale holes in model lipid membranes and cause dye leakage in cell culture experiments at concentrations in the range of 200 nM [137,138,139,140,141,142]. As a reference, this concentration is lower than the 1 mM concentration cited above for the CPP. Nevertheless, a variety of studies have demonstrated that cationic drug delivery systems (including CPPs) can buffer endolysosomal acidity. This has been termed the “proton sponge” effect [143], and it has been proposed that due to lysosomal swelling and rupture [144,145,146] this is important for allowing an efficient delivery. 

In general, the toxicities of dendrimers have been found to be dependent on the charge state of the dendrimer backbone and the peripheral groups [142]. Therefore, it is of interest to evaluate the potential toxicities of dendrimers with peripheral guanidines. Some studies have been carried out towards this goal. For example, 4th generation PPI based dendrimers were toxic with or without guanidinium groups, both exhibiting relative cell viabilities of approximately 50% at concentrations of 30 μg・mL^-1^ (after 24 h) [88]. The guanidine functionalized dendrimers were only slightly more toxic. Similar results were also obtained for melamine based dendrimers [92]. The latter were also hemolytic with a relative hemolysis of 50% (after 24 h) below 10 μg・mL^-1^. A 5th generation PPI dendrimer bearing 64 guanidine moieties was significantly more toxic (10 μM for 50% cell viability after 6 days) than the PPI with primary amine groups (40 μM for 50% cell viability after 6 days) [91]. Guanidine functionalized PPI dendrimers showed marked dose dependent cytotoxic effects as well as generation effects meaning that the number of guanidine groups was correlated to the toxicity [86,87]. A second generation dendrimer was consequently less toxic with HeLa and 293 cells, and even with only 8 guanidines, was efficiently internalized by the cells without showing toxicity [85]. Another interesting finding was that the toxicity was correlated to the efficiency of the internalization of polyamide based dendrimers when the effects of spacers with different degrees of flexibility were studied [29]. Nevertheless, other polyamide-based guanidinylated dendrimers were not toxic during the internalization [26,95]. Thus far there is only one example demonstrating the use of a dendritic guanidines *in vivo* and no toxicity was detected. This work involved carbamate based dendrons, which were successfully used for transfection in mice [98,99].

In some cases, it has been possible to reduce the toxicities of otherwise toxic cell penetrating dendrimers. Studies have focused on the use of lower generation dendrimers [85,86] when these lower generations have exhibited sufficient cell penetration. Another option has been to decorate the surface with two functionalities such as guanidines for cell uptake and hydroxyls for biocompatibility [88]. However a decrease in cell uptake was observed in this case. A third option was recently introduced using the PAMAM backbone, with or without the guanidines. The peripheral primary amines were first functionalized to provide a hydroxylated PAMAM dendrimer and then this dendrimer was functionalized with arginine and used for cell internalization with increased biocompatibility [81,82].

Overall, it is difficult to quantitatively compare the toxicities of CPPs with guanidinylated dendrimers as they have not been measured side by side on the same cell lines using the same assays. Such studies will be pertinent in future work in this field. However, based on the successful use of dendrimers in other biomedical applications and the preliminary results described above, it appears that it should be possible to develop relatively non-toxic cell penetrating dendrimers. The key to this success will be the choice of a dendrimer backbone that is preferably uncharged and non-toxic such as the polyester or polycarbamate, as well as careful tuning of the hydrophobic/hydrophilic balance and minimizing the number of guanidines to that required for activity.

## 7. Conjugation of Guanidine Functionalized Dendrimers to Cargo

**Figure 14:**
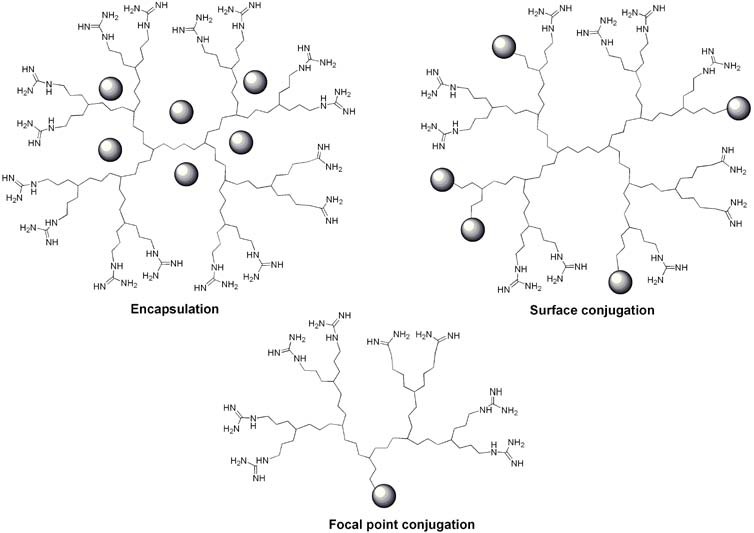
Guanidine functionalized dendrimers and their association with cargo: (a) encapsulation (b) surface conjugation (c) focal point conjugation.

Interest in CPPs rapidly increased when it became evident that they were able to act as vectors for the delivery of other molecules such as proteins [147,148,149,150], peptides [149,151,152,153], nucleic acids [154,155,156,157,158,159,160], imaging agents [161,162,163], nanocarriers [164,165,166,167,168], liposomes [164,169,170,171], and several small molecules [33,172,173,174]. Many molecules with anticancer, anti-inflammatory or antimicrobial activities have been successfully associated with dendrimers, either covalently or noncovalently [175]. First of all, dendrimers are particularly well-suited to encapsulate/complex many small molecules [39,176] (Figure 14a). 

These cargos can physically interact with dendrimers through either the encapsulation into void spaces, the association with surface groups, or a mixture of both. Driving forces for these interactions are hydrogen bonding, van der Waals interactions, and electrostatic attractions between opposite charges on the dendrimers and the cargos [177]. While the complexation of molecules within guanidinylated dendrimers has been investigated much less extensively, studies carried out thus far have yielded highly promising results. For example, guanidinylated PAMAM dendrimers have been used to encapsulate macromolecules such as DNA and RNA [79,80,81,82,83,85,88] for transfection applications. The conclusion of these studies was that guanidinylated PAMAM dendrimers were highly potent for transfection, even with vulnerable cells. For mechanistic studies, Paleos and coworkers have encapsulated betamethasone derivatives as dye molecules into guanidine functionalized dendrimers, but these molecules are also commonly used as anti-inflammatory agents [89].

**Figure 15:**
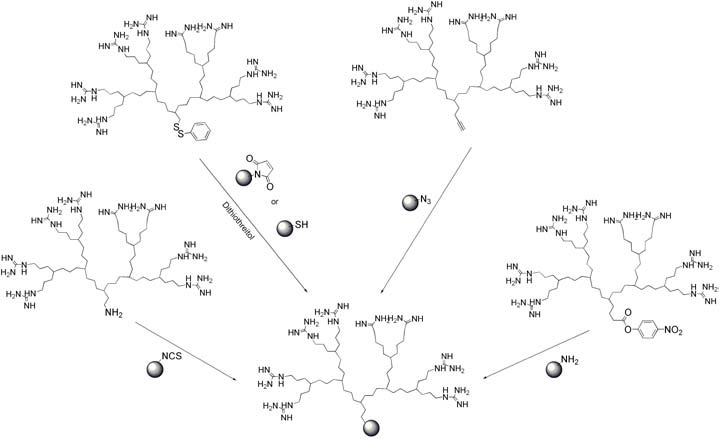
Conjugation to cargo via the dendron focal point.

Alternatively, different approaches have been developed to covalently conjugate the cargo. The first possibility is to conjugate cargo molecules to the dendrimer periphery (Figure 14b). This approach is often chosen [133] and has for example been used to synthesize polyester dendrimers as carriers of the anticancer drug doxorubicin [134] or to produce the dendrimer-based microbiocide VivaGel^TM^, which is in clinical trials for the prevention of HIV or HSV-2 (genital herpes) transmission [136]. With guanidinylated dendrimers this approach has also begun to be explored. For example, the partial reaction of nucleophilic surface functionalities on the dendrimer with activated fluorescein dye molecules (FITC), enabled the evaluation of the cellular internalization of the conjugate [84,86,87]. The second possibility was to couple dendrons to cargo molecules at their focal points (Figure 14c, Figure 15).

For example, dye molecules have been coupled with focal point amine groups of several different dendrons in order to study their cell uptake [26,29,95]. In addition, by using the focal point approach, Goodman and coworkers developed a coupling using a disulfide linker that enabled the conjugation of green fluorescent protein (GFP) to a dendritic guanidine [26]. Interestingly, the replacement of the low molecular weight fluorescein dye molecule with GFP did not change the translocation activity of their dendron. A disulfide linker was also used to couple dendritic guanidines to antibodies [97] and to polymer nanoparticles [96]. These conjugates were efficiently internalized by mammalian cells. For MRI applications, a polyester-based dendritic guanidine with a focal point alkyne was conjugated to azide functionalized Fe_3_O_4_ nanoparticles *via* a Cu(I) catalyzed “click” reaction [94]. This was demonstrated to enhance the cell uptake of the nanoparticles relative to control unfunctionalized nanoparticles, thus increasing the ability to detect the cells by MRI. For transfection applications, oligocarbamate-based dendrons were coupled to oligonucleotides [98,99]. In order to accomplish this, the dendrons were first functionalized to bear activated esters at their focal points, with which amines were subsequently reacted. This ‘dendrimeric octa-guanidine’ was highly effective to restore the dystrophin expression in both skeletal and cardiac muscles in the *dystrophic mdx mice*, as was the case with a CPP [178]. Moreover, Morcos et al. highlighted that this dendrimeric octaguanidine was highly potent for tissue penetration [98].

All of these results indicate that guanidinylated dendrimers can be successfully conjugated to a wide variety of cargos ranging from small molecules to biomacromolecules and nanoparticles. While much of this work is recent, it appears thus far that the dendrimers significantly enhance the cell uptake of their cargo, despite sizes approaching the tens of nanometres range. In addition, the controlled and step-wise nature of dendrimer chemistry enables the preparation of well-defined conjugates, particularly using the focal point approach. Future work in this area will likely involve the optimization of conjugation methods, the selection of the best transporters, and further biological studies of the conjugates. 

## 8. Conclusions and Perspectives

Dendrimers and dendrons bearing multiple peripheral guanidine moieties are promising analogues of CPPs. Although this is a new field with much of the progress occurring over the last several yeras, the syntheses of dendrimers is a sufficiently well developed field that a diverse range of dendrimer backbones can be readily prepared on relatively large scales and several reagents have been demonstrated to efficiently introduce guanidines to their peripheries. The many different available dendrimer backbones and linkers have allowed structure-activity relationships to be explored. This research has revealed that guanidine functionalized dendrimers can penetrate cells as efficiently as the HIV Tat_49-57 _peptide and that the incorporation of longer linkers into the dendrimer structure is important for this activity. It may also play a role in determining the subcellar localization of the transporter, an aspect that requires further study. In addition, the ability to tune the dendrimer’s generation has allowed the effect of varying the number of peripheral guanidine groups to be easily explored and it has generally been found that the optimal number is between 6 and 8. While higher numbers of guanidines lead to stronger interactions with membranes, the larger sizes of these molecules can make them more challenging to internalize and can lead to increased toxicity. 

The covalent and non-covalent conjugation of guanidine functionalized dendrons to biologically relevant cargo is an area that is just beginning to be developed. Thus far, some exciting results have emerged demonstrating that dendritic guanidines can facilitate the cellular internalization of even large cargo such as DNA, proteins, and nanoparticles. Nevertheless, cell viability upon internalization needs to be confirmed for these promising systems by the use of functional assays that require cell survival, such as the delivery of Cre recombinase [105,106]. Moreover, most of the research concerning guanidinylated dendrons has been carried out *in vitro*, so future efforts will need to focus on translating these results to animal models of disease. As part of this work, the *in vivo* toxicities and biodistribution behaviour of these molecules must be further explored before such systems can be applied in the clinic. It is known that cationic carriers, including CPPs can sometimes exhibit toxicity and research thus far has revealed that dendritic guanidines may not be an exception to this. Moreover, in order to be internalized by the target cells, the system must effectively reach the target site and evade undesired uptake by the reticuloendothelial system. The approaches for achieving this will be highly dependent on the target but may involve active targeting strategies and/or masking the cationic nature of protonated guanidines prior to their arrival at the target. Other promising applications involving the *in vivo* biodistribution and targeting of dendrimers, such as those outlined above, suggest that this work can be successful. Thus, in the future it is likely that the challenge in this field will be the conjugation of dendritic guanidines to a wider array of cargo aimed at specific therapies or imaging applications. In addition, the *in vitro* and *in vivo* efficacies and biocompatibilities of these systems must be explored. Nevertheless, significant progress has been made in this field during a relatively short time period and the results thus far demonstrate that dendritic guanidines are promising molecules for intracellular delivery and are worthy of further attention.
